# Torsion of the caudate lobe of the liver and concurrent necrohemorrhagic typhlocolitis in a zoo-housed Patagonian mara

**DOI:** 10.1177/10406387241248594

**Published:** 2024-05-04

**Authors:** Catherine Wilson, Steven J. Philp, Katherine Hughes

**Affiliations:** Department of Veterinary Medicine, University of Cambridge, Cambridge, UK; International Zoo Veterinary Group UK, Keighley, UK; Department of Veterinary Medicine, University of Cambridge, Cambridge, UK

**Keywords:** liver, Patagonian mara, pathology, *Salmonella*, torsion, typhlocolitis

## Abstract

Liver lobe torsion has been reported in many species, with frequent reports in rabbits. Here we describe caudate liver lobe torsion and concurrent necrohemorrhagic typhlocolitis in a Patagonian mara (syn: Patagonian cavy, Patagonian hare, *Dolichotis patagonum*). Following acute death, postmortem examination findings included torsion of the hepatic caudate process, which had fibrous adhesions to the pancreas indicating chronicity. The cecal apex and proximal 30 cm of colon had regionally reddened serosa and diffusely roughened and reddened mucosa with brown-red and granular luminal contents. Key histologic findings included massive necrosis of the torsed hepatic caudate lobe, consistent with infarction, necrotizing hepatitis in remaining areas of liver, necrohemorrhagic typhlocolitis, adrenocortical necrosis and hemorrhage, and renal tubular degeneration and necrosis with tubular casts. Bacterial culture of cecal contents yielded pure growth of *Salmonella* spp. Death was attributed to toxemia or bacteremia resulting from *Salmonella* spp. infection, as the hepatic lobe torsion appeared chronic. It was undetermined if the liver lobe torsion predisposed to gastrointestinal compromise and infection. Patagonian maras have some anatomical similarities to rabbits and are highly cursorial, not dissimilar to hares, *Lepus* spp. We speculate that these characteristics may increase the likelihood of hepatic caudate lobe torsion in this species.

The Patagonian mara, *Dolichotis patagonum*, also referred to as the Patagonian cavy or Patagonian hare, is a large rodent with an adult body mass of 7–9 kg. A member of the family *Caviidae*, this species is relatively hardy and thus popular in zoologic collections in countries with temperate climates.^9^ Herein, we report pathologic findings in a case of hepatic caudate lobe torsion and concurrent necrohemorrhagic typhlocolitis in a zoo-housed Patagonian mara. The potential relationship between these 2 conditions in this mara is considered.

A 9-y-5-mo-old intact male Patagonian mara was found dead in its enclosure at a zoologic park. No signs of injury or illness had been noted the previous day. The mara was the only affected animal in a group of 4 maras.

On external examination, the mara was considered lean, weighing 6 kg, with a small amount of subcutaneous fat. Body condition was considered to potentially indicate underlying disease. The caudate process of the liver was torsed at the hilum, variegated red and off-white, friable and “chalky” on cut surface (Fig. 1), and firmly adhered to the pancreas by fibrous adhesions. The remaining hepatic parenchyma was brown, with red areas of irregular size and shape and occasional depressed gray areas, all <5 mm diameter. Regionally, the apex of the cecum had a reddened serosa and a roughened, reddened mucosa (Fig. 2). The remainder of the cecum had a gray-green mucosa and gray serosa. The cecum and proximal 30 cm of the colon contained brown-to-red, granular melenic content. This segment of colon had a gray serosa and a reddened, roughened mucosa (Fig. 3). The cecum and colon also contained numerous off-white nematodes ~10 mm long and 0.5 mm diameter. Bilaterally, the kidneys had ~2–3 mm, well-demarcated red foci that were visible on the subcapsular surface and extended into the parenchyma. Bilaterally, the adrenal cortices had up to 2-mm dark-red foci. The remaining adrenal cortex was dark-pink.

**Figures 1–7.**
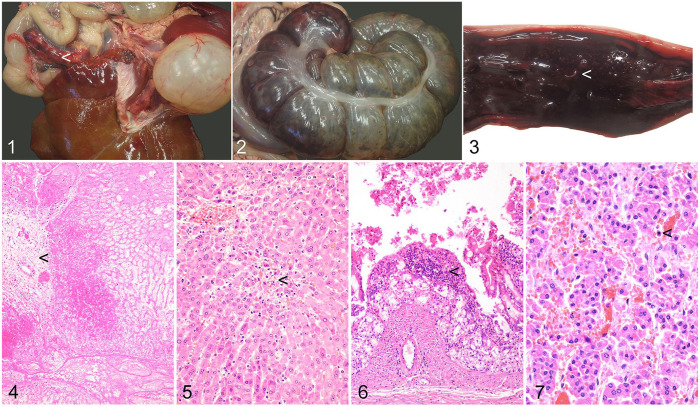
Torsion of the caudate lobe of the liver and coincident necrohemorrhagic typhlocolitis in a Patagonian mara (*Dolichotis patagonum*). **Figure 1.** The caudate process of the liver is torsed, multifocally red and off-white, with a “chalky” texture (arrowhead). **Figure 2.** In a locally extensive focus, the cecal serosa is red. Moderate numbers of nematodes are apparent. **Figure 3.** The colon mucosa is roughened and red. Nematodes are present (arrowhead). **Figure 4.** Coagulative and lytic parenchymal necrosis of the torsed caudate lobe of the liver. Portal areas are fibrotic (arrowhead). **Figure 5.** Randomly distributed, necrotizing hepatitis (arrowhead) in the remaining hepatic lobes. **Figure 6.** In the cecum and colon, mucosal hemorrhage and necrosis are accompanied by small numbers of inflammatory cells (arrowhead). **Figure 7.** Small hemorrhages are present in the adrenal cortex. Occasional necrotic cortical epithelial cells are evident in these foci (arrowhead). H&E (Figs. 4–7).

Microbiologic culture of cecal contents yielded growth of *Salmonella* sp. that was not further speciated. Samples of liver, pancreas, small and large intestines, cecum, kidneys, spleen, adrenal gland, lungs, and heart were fixed in 10% neutral-buffered formalin for 24 h, embedded in paraffin, and routinely processed to generate 4-µm thick, H&E-stained histologic sections.

The most significant microscopic finding was chronic, diffuse, severe coagulative and lytic parenchymal necrosis of the caudate lobe of the liver, consistent with infarction. Some portal areas were fibrotic, indicative of chronicity (Fig. 4). The remainder of the hepatic parenchyma had acute, moderate, multifocal and random, necrotizing hepatitis (Fig. 5). Fibrous tissue spanned the pancreas and hepatic caudate lobe forming firm adhesions.

Acute, segmental, marked necrohemorrhagic typhlocolitis was present, with small numbers of lymphocytes and plasma cells and fewer neutrophils extending multifocally into the submucosa. There were moderate numbers of intraluminal nematodes with morphology including an eosinophilic cuticle, a pseudocoelom with coelomyrian-polymarian musculature, and a reproductive tract containing very large numbers of bipolar plugged eggs, together consistent with *Trichuris* spp. The cecal mucosa had multifocal ulceration, and the lumen contained extensive hemorrhage (Fig. 6). Small hemorrhages were present in the adrenal cortex with occasional epithelial cell necrosis (Fig. 7). There was multifocal renal epithelial tubular degeneration and necrosis.

Our final morphologic diagnoses were torsion of the caudate lobe of the liver leading to infarction and subsequent pancreatic adhesions. There was also acute necrotizing hepatitis and necrohemorrhagic typhlocolitis, adrenocortical hemorrhage and cortical epithelial necrosis, and renal tubular degeneration and necrosis with tubular casts. We considered the cause of death to be toxemia or bacteremia resulting from *Salmonella* spp. infection. This likely led to necrotizing hepatitis, necrohemorrhagic typhlocolitis, and the lesions within the kidneys and adrenal glands. The caudate liver lobe torsion was interpreted as a separate process of longer chronicity. Intraluminal nematodes, morphologically consistent with *Trichuris* spp., and moderate portal hepatitis were diagnosed to most likely be incidental findings.

Liver lobe torsion has been rarely reported in many species of animal including the rabbit,^2,3,11,20^ horse,^1^ dog,^15^ cat,^15^ pig,^5^ Asian small-clawed otter,^18^ ferret,^17^ guinea pig,^4,19^ camel,^8^ and non-human primates.^14^ The definitive cause of liver lobe torsion is often unconfirmed although suggested predisposing factors include hepatic ligament absence, pathologic increase in size of abdominal organs (see below), or trauma.^15^ Parasitic or bacterial infections, or a hepatic mass such as an abscess or neoplasm, have also been implicated in some cases.^2,15^ Gastrointestinal infection leading to liver lobe torsion would seem very unlikely in our case given the chronic appearance of the hepatic lobe torsion compared to the acute nature of the lesions associated with the *Salmonella* spp. infection. It is not possible to confirm whether the 2 pathologic processes are connected, although it is possible that the necrotic hepatic lobe subsequent to torsion rendered the mara at increased susceptibility to the development of *Salmonella* spp. infection.

In species other than rabbits, it is suggested that torsion is commonly seen in the left lateral hepatic lobe due to the level of its separation from the other lobes and degree of mobility. By comparison, torsion of the caudate lobe is relatively frequently reported in rabbits, postulated to be due to the smaller, circular shape of this lobe, and the narrow point of attachment at the hilum.^2,3,11^ Patagonian maras are highly cursorial, not dissimilar to hares, *Lepus* spp.^9^ We speculate that this characteristic may increase the likelihood of caudate lobe torsion in this species. Although maras may be maintained in enclosures in zoologic collections, short bursts of activity during interactions with conspecifics may still occur. Studies examining the hepatic anatomy of the Patagonian mara are merited to better understand any species-specific predisposing factors.

No clinical signs were reported to affect this Patagonian mara prior to its death. The clinical signs of caudate liver lobe torsion in rabbits have been reported to be fairly nonspecific, including hyporexia or anorexia, lethargy, and decreased fecal production.^3,11^ These signs may be difficult to detect in a species that is a prey animal. Consistent with the histologic chronicity of the lesion, we, therefore, consider it plausible that the affected Patagonian mara had hepatic lobe torsion for some time prior to its death. This is also in line with a previous suggestion that hepatic caudate lobe torsion can be an incidental finding during postmortem examination of laboratory rabbits.^20^

In our case, the necrotizing hepatitis, necrohemorrhagic typhlocolitis, and renal and adrenal necrosis were attributed to the *Salmonella* spp. infection. In an analysis of clinical disease affecting a zoo-housed colony of maras in Mexico, bacterial infection caused 8 of 54 deaths within the colony, but none of these fatalities were attributed to infection with *Salmonella*.^12^ *Salmonella* spp. infection has been recorded at a zoologic garden in Japan where a number of Patagonian maras exhibited multi-organ system hemorrhage and necrosis attributed to *Salmonella enterica* subsp. *enterica* Enteritidis.^10^ Clinical signs of *Salmonella* infection within this cohort were reported as inappetence, difficulty in standing, and convulsions. Notably, the maras exhibited sudden or peracute death in the absence of diarrhea, consistent with our case.^10^

Some *Salmonella* spp. have a broad host range and can be zoonotic, both of which are particular considerations in a zoologic collection. The source of infection was not identified in our case, but *Salmonella* spp. can be detected in contaminated animal feed or water, the environment, and human or animal carriers. Clinical disease may be multifactorial, with stress also having a potential impact.^10,13^ Rodents have been implicated in bacterial disease transmission in zoologic collections.^6,7^ In our case, we suspect ingestion of *Salmonella* spp. from the environment, followed by subsequent infection, although the exact source of infection within the environment is not clear.

Our mara also had a moderate burden of *Trichuris* spp. nematodes. *Trichuris* spp. have been reported in zoo-housed Patagonian maras, and we consider the infection to be an incidental finding in this patient.^16^
